# Allophonic perception of VOT contrasts in Spanish children with dyslexia

**DOI:** 10.1002/brb3.2194

**Published:** 2021-05-21

**Authors:** Willy Serniclaes, Miguel López‐Zamora, Soraya Bordoy, Juan L. Luque

**Affiliations:** ^1^ Institute of Neuroscience and Cognition CNRS, UMR 8002 Université Sorbonne Paris Cité Paris France; ^2^ Unité de Recherche en Neurosciences Cognitives Centre de Recherches en Cognition et Neurosciences Université Libre de Bruxelles Bruxelles Belgium; ^3^ Departamento de Psicología Evolutiva y de la Educación Facultad de Psicología y Logopedia Universidad de Málaga Málaga Spain; ^4^ Departamento de Psicología Evolutiva y de la Educación Facultad de CC de la Educación Universidad de Granada Granada Spain

**Keywords:** allophonic perception, categorical perception, dyslexia, Spanish, voice onset time

## Abstract

**Introduction:**

Previous studies have evidenced a different mode of speech perception in dyslexia, characterized by the use of allophonic rather than phonemic units. People with dyslexia perceive phonemic features (such as voicing) less accurately than typical readers, but they perceive allophonic features (i.e., language‐independent differences between speech sounds) more accurately.

**Method:**

In this study, we investigated the perception of voicing contrasts in a sample of 204 Spanish children with or without dyslexia. Identification and discrimination data were collected for synthetic sounds varying along three different voice onset time (VOT) continua (ba/pa, de/te, and di/ti). Empirical data will be contrasted with a mathematical model of allophonic perception building up from neural oscillations and auditory temporal processing.

**Results:**

Children with dyslexia exhibited a general deficit in categorical precision; that is, they discriminated among phonemically contrastive pairs (around 0‐ms VOT) less accurately than did chronological age controls, irrespective of the stimulus continuum. Children with dyslexia also exhibited a higher sensitivity in the discrimination of allophonic features (around ±30‐ms VOT), but only for the stimulus continuum that was based on a nonlexical contrast (ba/pa).

**Conclusion:**

Fitting the neural network model to the data collected for this continuum suggests that allophonic perception is due to a deficit in “subharmonic coupling” between high‐frequency oscillations. Relationships with “temporal sampling framework” theory are discussed.

## Graphical abstract

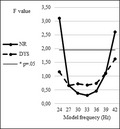

**Research highlights**

- Spanish children with dyslexia exhibit a general deficit in the perception of the voicing feature on three different VOT continua (ba/pa, de/te, and di/ti).
- These children also exhibit a higher sensitivity in the discrimination of allophonic features, but only for the stimulus continuum that was based on a nonlexical contrast (ba/pa).
- Fitting a neural network model to the data suggests that allophonic perception is due to a deficit in “subharmonic coupling” between high‐frequency networks.

## 1 INTRODUCTION

Developmental dyslexia is a disorder in which the ability to decipher written language is impaired despite the absence of other cognitive or sensory impairments (Peterson & Pennington, 2012). Dyslexia has a genetic basis, but the epigenetic factors that determine its expression remain under investigation. Various factors have been suspected to cause dyslexia, and most of these factors are related to deficits in one of the three components of written language: phonology, visual information, and phonovisual information (Serniclaes & Sprenger‐Charolles, 2015). The phonological deficit is thought to be the most common source of dyslexia (Saksida et al., 2016), and it most frequently emerges as a deficit in phonemic awareness (Melby‐Lervåg et al., 2012), that is, a deficit in conscious access to and manipulate phonemic representation (Shaywitz & Shaywitz, 2003). Converging evidence from different languages suggests that the phonological deficit in dyslexia is related to a weakness in phonemic perception (i.e., Noordenbos & Serniclaes, 2015). The aim of the present paper was to provide further insights on the nature of such deficit, getting new empirical evidences and interpreting them at the light of current theoretical models, as the temporal sampling framework (Goswami, 2011).

In the current study, we investigate the deficits associated with the perception of voicing contrasts between phonemes in Spanish school‐aged children. Previous studies showed that developmental dyslexia was related to a deficit in the categorical perception of phonemes and that such deficit arouse from the enhanced perception of allophones, that is, subphonemic units without phonological content (for a review, see Serniclaes, 2018). Here, we investigate the allophonic perception of voicing contrasts in a fairly large sample of Spanish children. The first aim of the study was to extend previous evidence about allophonic perception that was obtained with French children with dyslexia (Serniclaes et al., 2004). Second, we used the neural evidence in the literature as a framework to produce a mathematical model of allophonic perception. Considering the current temporal sampling framework, we explored how allophonic perception deficit could be explained by a coupling deficit between neural oscillators.

### 1.1 Allophonic perception

Different studies have evidenced the implications of speech perception deficits for reading acquisition (e.g., O’Brien et al., 2018; Snowling et al., 2019). Previous studies have shown that children suffering from developmental dyslexia have a deficit in categorical perception of speech sounds, characterized by a weaker convergence between discrimination and identification of sounds (Werker & Tees, 1987; for a review: Noordenbos & Serniclaes, 2015). Further, it was shown that dyslexics exhibit weaker discrimination between phoneme categories and also *better* discrimination within categories (Serniclaes et al., 2001). Such enhanced sensitivity to acoustic differences within phoneme categories has been related to a specific mode of speech perception that is based on “allophonic” features, that is, universal (language‐independent) properties that are contextually variable cues for phonemic distinctions in a given language (Serniclaes et al., 2004). Recent studies evidenced the implications of allophonic perception for reading and meta‐phonological skills, that is, those involved in the conscious manipulation of phonemes and other phonological units (Hämäläinen et al., 2018; Li et al., 2019).

Phonemes are classically defined as “bundles” of distinctive features (Chomsky & Halle, 1968; Jakobson et al., 1952), and phoneme perception is essentially a matter of binding different features that are scattered over time in the speech signal. Normally, several different allophonic features are integrated to perceive contextually invariant phonemic categories. With allophonic perception, these features are not integrated, and they give rise to within‐category percepts. Behavioral studies showed that dyslexia is associated with an enhanced sensitivity to allophonic variants of different phonemic features, such as place of articulation (in Dutch: Noordenbos et al., 2012a) and voicing (in French: Bogliotti et al., 2008; Serniclaes et al., 2004).

### 1.2 Allophonic perception of voice onset time categories

The voicing feature is used in almost all languages to separate different categories of stop consonants. Voicing perception in initial stops is mainly based on voice onset time (VOT), which is the time interval between the onset of voice (laryngeal periodic vibrations) and the release of vocal tract closure (Lisker & Abramson, 1964; Lisker et al., 1977). The interest of VOT for phoneme perception is that it specifies the temporal relationship between voicing (the onset voiced vibrations) and other distinctive features (e.g., manner and place of articulation as indexed by burst and formant transitions). In this sense, VOT perception offers some insights to understanding phoneme perception.

The vocal tract can reliably produce at most three VOT categories in a given language, characterized by negative VOT, short positive VOT, and long positive VOT (Abramson, 1977). For the languages that use all three different VOT categories, the perceptual boundaries between these categories are located at about −30‐ and +30‐ms VOT (e.g., in Thai; Abramson & Lisker, 1970). For the languages that only use two different categories, the VOT boundaries between these categories are located at about either +30‐ms VOT (e.g., in English; Abramson & Lisker, 1970) or 0‐ms VOT (e.g., in Spanish and French; Abramson & Lisker, 1973; Serniclaes, 1987).

Different sources of evidence indicate that VOT boundaries that are located at about −30 ms and +30 ms are universal. Such boundaries were evidenced in young infants before six months of age, using discrimination responses to stimuli varying along a VOT continuum (for a review, see Hoonhorst, Colin, et al., 2009). The universal sensitivity to VOT contrasts around 30 ms was also evidenced with neural data. Different studies have evidenced neural response peaks in response to acoustic differences across 30‐ms VOT, in the cortex of both humans and monkeys (Steinschneider et al., 1995, 1999). The neural sensitivity to the 30‐ms VOT boundary is present even in languages where the phonemic boundary is located at 0‐ms VOT (e.g., in French: Hoonhorst, Serniclaes, et al., 2009).

According to the allophonic theory, the categorical perception deficit in dyslexia results from a lack of coupling between universal features, giving rise to an increased sensitivity to within‐category allophonic contrasts (Serniclaes et al., 2004). Concerning voicing perception in Spanish, and other languages (such as French) with a 0‐ms VOT boundary, people with dyslexia should be sensitive to universal allophonic boundaries, that is, those located at ±30‐ms VOT. Previous data collected in French showed that children with dyslexia indeed exhibit an enhanced sensitivity to the −30‐ms boundary (Bogliotti et al., 2008; Serniclaes et al., 2004). However, such enhanced sensitivity could not be evidenced for the +30‐ms boundary with the VOT continua used in these studies.

Categorical *precision*, defined as the accuracy of the phoneme boundary, can be used as proxy for assessing allophonic perception. Categorical precision is inversely related to allophonic perception, but it also depends on the intrinsic sensitivity to phonemic contrasts, independently of the concurrent sensitivity to allophonic ones. The degree of categorical precision depends on the age/grade for both normal‐reading children (Hoonhorst et al., 2011; Medina et al., 2010) and for children with dyslexia (Noordenbos et al., 2012a). However, adults with dyslexia still present a categorical precision deficit compared with normal‐reading adults (Noordenbos et al., 2013).

### 1.3 Neural‐based mathematical model of VOT perception

As we have above mentioned, young infants showed a universal sensitivity to the 30‐ms VOT boundaries, suggesting the existence of some basic neural mechanism, possibly oscillators operating at a frequency of about 33 Hz (30‐ms period) in the low‐gamma band. This contention is also supported by empirical evidence showing that intracranial stimulation with 40‐Hz modulation affects the precision of the VOT boundary in the perception of a German voicing contrast (Rufener et al., 2016), using a continuum on which this boundary is precisely located at 30 ms (Zaehle et al., 2007). A subsequent study showed that intracranial stimulation with 40‐Hz modulation improves VOT perception in dyslexics (Rufener et al., 2019).

**FIGURE 1:**
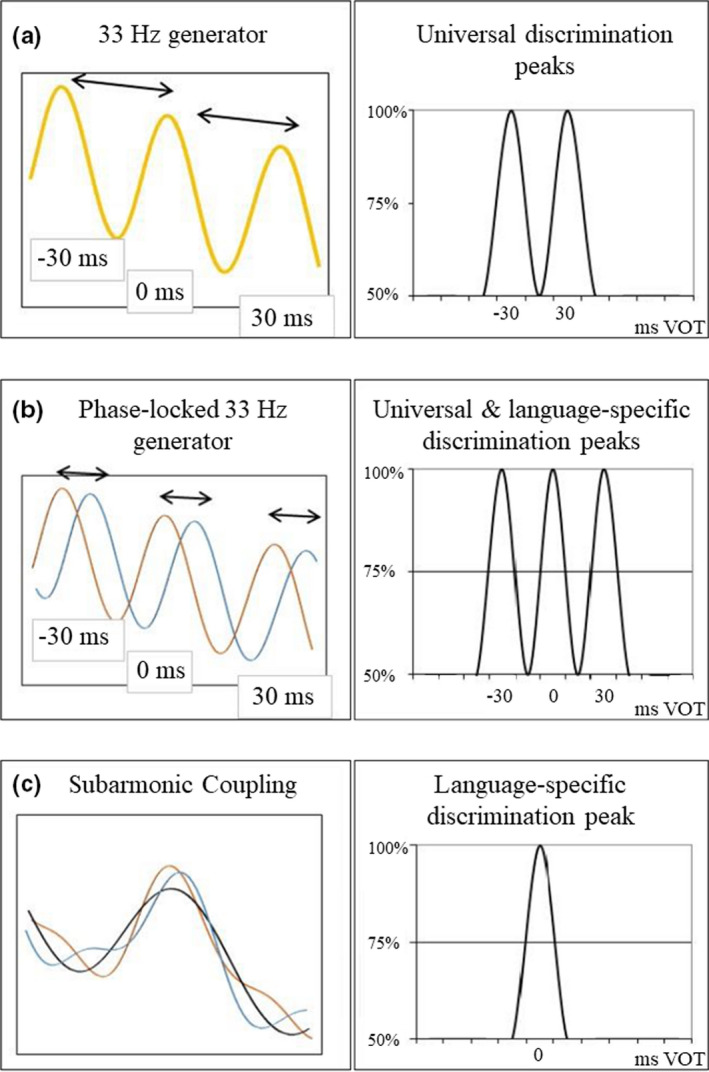
Developmental patterns of voice onset time (VOT) perception and behavioral implications. Right side, neural settings; Left side: behavioral implications on discrimination responses (respectively). (a) Single 33‐Hz oscillator; (b) phase locking between 33‐Hz oscillators (blue and yellow lines); and (c) subharmonic coupling between 33‐Hz oscillators (black line)

Obviously, the sensitivity to the VOT boundary is somehow related to neural oscillators in the low‐gamma range, and dyslexia is related to a dysfunction in the way these oscillators operate to detect VOT differences. In order to better understand the nature of such dysfunction, we need to specify the mechanism by which gamma oscillators capture the temporal relationships between the acoustic events that shape VOT perception, that is, the noise burst that signals a stop consonant and the onset of voice. Figure 1 presents the different neural models of VOT perception and their implications for the discrimination of stimuli varying along a VOT continuum. The simplest model (Figure 1a) postulates that the burst and voice onset are detected by gamma oscillators that are perfectly synchronized, that is, with zero phase difference. Such mechanism accounts for the discrimination peaks located at ±30‐ms VOT, both the universal peaks evidenced in young infants (Hoonhorst, Colin, et al., 2009) and those evidenced in languages that use three different VOT categories (e.g., Thaï: Lisker & Abrmason, 1970). However, a mechanism based on synchronized gamma oscillators has several limitations. Firstly, it cannot account for the 0‐ms discrimination peak in languages such as Spanish (Abramson & Lisker, 1973). A second limitation of such mechanism is that it cannot account for the contextual flexibility of VOT boundaries within all languages. The ±30‐ms boundaries, as well as the 0‐ms one, correspond to the mean values of the boundaries that otherwise change as a function of the phonemic context (Lisker et al., 1977). Such limitations are overcome by a mechanism that uses a dephasing between the oscillators that detect burst and voice onset (Figure 1b). This mechanism accounts for the 0‐ms discrimination peak in languages such as Spanish. It can also account for the contextual flexibility of the VOT boundaries in all languages, a larger dephasing producing a larger boundary shift. However, a mere dephasing between oscillators would give rise to three different discrimination peaks (Figure 1b), including those that do not correspond to a phonemic boundary in a given language. In other words, such mechanism would give rise to both phonemic and allophonic (within‐category) discrimination peaks, which is exactly the perceptual profile that is expected for people with dyslexia.

In order to explain normal speech perception, the dephasing mechanism has to be completed with a damping process that inhibits the detection of temporal differences outside the boundary region. The damping might be achieved by a subharmonic coupling between oscillators, a mechanism by which the phase relationships between oscillators are controlled by entrainment to an oscillator operating at a subharmonic frequency (Yang et al., 2016). Subharmonic coupling has been evidenced for a wide range of phenomena in the neurocognitive domain (Langdon et al., 2011; Roberts & Robinson, 2012). The implications of subharmonic coupling on the discrimination peaks in a language with a 0‐ms VOT boundary are illustrated in Figure 1c. A ~17‐Hz network (more precisely 16.67 Hz), the binary subharmonic of ~33 Hz, controls the phase difference between the two ~33‐Hz oscillators, resulting in a single (between‐category) discrimination peak.

### 1.4 The present study

The main empirical objective of the present study was to evidence allophonic perception in a fairly large sample of about 100 Spanish children with dyslexia and 100 control children at two different school levels (Grades 2 and 4). Identification and discrimination data were collected for synthetic sounds varying along three different VOT continua (ba/pa, de/te, and di/ti). Previous investigation has shown that evidencing allophonic perception with behavioral data is difficult and might depend on the stimulus continuum. Concerning voicing, allophonic perception was evidenced in French with ba/pa and ga/ka and do/to continua (Bogliotti et al., 2008; Serniclaes et al., 2004) but not with a də/tə continuum (Zoubrinetzky et al., 2019). There are no obvious reasons for such discrepancies. However, differences in place‐of‐articulation and vocalic context, two factors that play a role in voicing perception (Lisker & Ambramson, 1967; Serniclaes, 1975), might play a role. Differences in lexical status between the continua might also be the culprit. The də/tə contrast is much more frequent in French compared with those of the ones used in the other studies.^1^ Our motivation for using of three different continua in the present study was to increase the possibility to evidence allophonic perception. The continua that were used vary in place of articulation, vocalic context, and lexical status. Accordingly, the fact that categorical precision was lower for the ba/pa continuum compared with the two d/t continua can be attributed to a difference in lexical status. Both /ba/ and /pa/ are pseudowords in Spanish, whereas the /de/, /te/, /di/, and /ti/ are real words. We thus took account of the possible factors that might affect evidencing allophonic perception. As these factors do not vary orthogonally, their perceptual effects could not be tested independently. But this was not our aim. Our aim was to maximize the chances to evidence allophonic perception with only three continua. Using more continua (there are 18 possible combinations between place, voicing context, and lexical status) would have been a formidable task.

Besides evidencing allophonic perception, the other main objective of the present study was to evidence possible links between such allophonic sensitivity and a lack of subharmonic coupling between generators in the low‐gamma range. A secondary empirical objective was to assess between‐group differences in categorical precision, which are somewhat related to allophonic perception but are less specific.

Beyond allophonic deficit has shown as a robust empirical result, and it is necessary to understand its nature at the neural level. Different neural oscillation bands and their couplings have been proposed as speech recognition brain mechanisms (Hickok & Poeppel, 2007). The cross‐frequency coupling study of brain activity is a complex task, so some hypotheses are advanced by computational simulations, guiding the exploration of posterior brain studies (i.e., Hovsepyan et al., 2020). Accordingly, we will explore which oscillation bands and which couplings between them might account for an allophonic perception deficit.

## 2 METHOD

### 2.1 Participants

Two hundred and four children from 14 schools in southern Spain were selected from an initial sample of 1,158 participants of children of 2nd and 4th grade (see Table 1). The TECLE test (Marín & Carrillo, 1997) was used to select children with clear reading difficulties, as well as children with clearly average or superior reading skills for their age. TECLE is a forced‐choice sentence completion test (Marín & Carrillo, 1997). TECLE consists of 64 sentences that have a missing word. To fill the gap, the participant had to choose the correct answer among four orthographically similar options. The complexity of the sentences increased as the reader progresses through the test. Children were asked to read silently and complete as many sentences as they could in five minutes, and the results were scored in number of correct responses. Here, we used TECLE data collected in a sample of 1,186 s‐ and fourth‐grade students (562 and 624 participants, respectively) from 14 schools in the province of Malaga in Spain (Bordoy, 2015, p.: 88–89) as a reference. In the present study, the 2nd‐ and 4th‐grade normal reader (NR) groups included children with efficiency measures between the mean and the mean +1.0 *SD* of the TECLE test scores. The two dyslexic groups (DYS) were selected for the 2nd and 4th grade and included children whose performance on the TECLE test was below −1.5 *SD*. The 4th‐grade dyslexic group was matched with the reading level of the normal‐reading children in the 2nd grade. Thus, the 2nd‐grade NR was used as a reading‐level control group when necessary. Nonverbal intelligence was measured with the RAVEN test (Raven et al., 1996) to confirm that all children were in the typical range. Special care was given to remove from the sample children who had neurological, auditory perception, visual perception, sensory‐motor deficits, oral language deficits, or other problems used as exclusion criteria for a specific learning disability diagnosis. Although we did not have specific diagnoses, this information was obtained from interviews with their teachers, psychoeducational reports from schools, and the informed consent from parents, in which they reported whether the children had any specific type of problem that could alter this investigation. Of the 204 children who participated in the study, there were 97 children with dyslexia (hereafter DYS), among which 57 were in 2nd grade and 40 were in 4th grade, and 107 normal‐reading controls (hereafter NR), among which 64 were in 1st grade and 43 were in 2nd grade (see Table 1).

**TABLE 1:** Sample characteristics

Groups	*N*	Age in years (*SD*)	TECLE % success rate means (*SD*)
2nd‐Grade DYS	57	7.5 (0.4)	9.80 (6.73)
2nd‐Grade NR	64	7.8 (0.5)	27.44 (9.15)
4th‐Grade DYS	40	9.6 (0.5)	21.92 (8.06)
4th‐Grade NR	43	9.9 (0.3)	55.58 (6.99)

### 2.2 Stimuli

Three VOT continua were used, corresponding to different contrasts between stop consonants: ba/pa, de/te, and di/ti. Each continuum was composed of eleven synthetic stimuli differing in VOT, increasing from −50 ms to +50 ms in 10‐ms steps. The postrelease segment (i.e., positive VOT plus voiced vocalic segment) was constant, but the total duration increased as a function of negative VOT. This stimulus design was chosen because changes in total duration, including negative VOT, are less audible than changes in the postrelease segment.

The stimuli were generated by parallel formant synthesis using software implemented by Carré (CNRS, France, http://pagesperso‐orange.fr/ren.carre/index.htm). The stimuli with −50, 0, and positive VOT were individually synthesized. Those with the remaining negative VOT values were obtained by editing the prevoicing segment in the −50‐ms VOT stimulus. Negative VOT was synthesized with periodic energy (60 dB), F1 bandwidth of 50 Hz, and F2 and F3 bandwidths both of 600 Hz. Positive VOT was synthesized with periodic energy (30 dB), with a F1 bandwidth of 600 Hz, and F2 and F3 bandwidths of 70 and 100 Hz, respectively. The voiced vocalic segment was synthesized with periodic energy (60 dB) and with F1, F2, and F3 bandwidths of 50, 70, and 100 Hz, respectively. The F0 was fixed to 120 Hz. The formant transitions lasted 24 ms, and the stable vocalic portion lasted 180 ms. The duration of the postrelease part of the stimuli was constant (204 ms), and the total duration depended on negative VOT, which varied between 50 and 0 ms.

For the ba/da continuum, the starting frequencies of F1, F2, and F3 transitions were of 200, 2,100, and 3,100 Hz, respectively. The end values of the transitions were fixed at 500, 1,500, and 2,500 Hz, respectively, for F1, F2, and F3. For the de/te continuum, the starting frequencies of F1, F2, and F3 transitions were of 200, 2,100, and 3,100 Hz, respectively. The end values of the transitions were fixed at 500, 1,500, and 2,500 Hz, respectively, for F1, F2, and F3. For the di/ti continuum, the starting frequencies of F1, F2, and F3 transitions were of 200, 2,100, and 3,100 Hz, respectively. The end values of the transitions were fixed at 500, 1,500, and 2,500 Hz, respectively, for F1, F2, and F3.

#### 2.2.1 Procedure

E‐Prime 1.02 experimental software was used for presenting the stimuli and collecting the responses. The stimuli were presented over noise‐canceling headphones, and responses were given on a computer keyboard. The procedure comprised four successive stages: an explanation of the procedure of the task, a five‐minute trial session, the identification test, and the discrimination test. During the explanation of the procedure, the participants were instructed on how to deliver the identification and discrimination responses with the continuum endpoints. Before each task, a five‐minute trial session with only the endpoint stimuli of each continuum was administered in which the subject had to correctly answer 75% of the items. If the child failed the test, the experimenter explained the instructions again to make sure she or he understood them correctly. If they failed a second time, they were excluded from the experiment (it only happened with eleven participants, i.e., about 4% of the sample).

For the identification test, participants had to identify the stimuli, as either /b/ or /p/, or /d/ or /t/, depending on the continuum. Each stimulus was presented eight times in a pseudorandom order (88 trials in total). Responses were given by pressing a different key each covered by a colored patch with different printed letters (B/P for the b/pa continuum and D/T for de/te and di/ti continua), on a QWERTY computer keyboard.

For the discrimination test, participants were asked to indicate whether a pair of stimuli were the same or different. “Different” pairs were stimuli that differed by 20‐ms VOT (AX format) (e.g., −50‐ms VOT followed by −30‐ms VOT, or −30‐ms VOT followed by −50‐ms VOT). “Same” pairs were two stimuli of identical VOT (e.g., two times −50‐ms VOT, or two times −30‐ms VOT). There were 18 “different” pairs (nine stimulus combinations in two orders) and 11 “same” pairs for each continuum. Each pair was presented eight times in a pseudorandom order (248 trials in total). Answers were given by pressing either the “M” key for same (i.e., “mismo” in Spanish) or the "D" key for different (i.e., “diferente” in Spanish). The keys are covered by a color patch (blue for “same,” yellow for “different”), with printed M or D letters, also on a QWERTY keyboard.

The total procedure took approximately 30 min. All the participants completed the identification test first, then the discrimination test. In order to avoid learning effects, the different continua were counterbalanced.

### 2.3 Data processing

All the analyses were performed with SPSS‐25©.

#### 2.3.1 Identification data

The precision of categorical boundaries was assessed on the basis of both the slope (Simon & Fourcin, 1978) and the asymptotes of the identification curves (for details, see Noordenbos et al., 2012a). The asymptotes correspond to the identification responses at the endpoints of the stimulus continuum, and they should normally be close to either 0 or 100%. Shallower slopes and/or asymptotes closer to 50% indicate lower precision. The boundary, slope, and asymptotes of the identification function were assessed for each subject using a 4‐parameter logistic model (Richards, 1959), with the identification response as the dependent variable and the stimulus as the independent variable. With asymptotes close to either 0 or 100%, Richards’ model (Equation 1) resumes to the classical logistic function (Equation 2).

(1) $$P\left(\text{responses}\right)=K1+\left(K2‐K1\right)/\left[e^{y}/\left(e^{y}+1\right)\right].$$

(2) $$\text{with K}1=0\;\text{and K}2=1:P\left(\text{responses}\right)=e^{y}/\left(e^{y}+1\right).$$

Equations 1 and 2: *y* = *I* + *S* × stimulus values (*I* = intercept; *S* = slope of the identification function); boundary = *I*/*S*; K1 = lower asymptote; K2 = upper asymptote. The interest of Richards’ model (Equation 1) is that it captures differences not only in the slope of the identification function, but also in its asymptotic values. Estimates of the parameters of Richards’ model were obtained by nonlinear regression. The slope, boundary location—hereafter “boundary”—and difference between the upper and lower asymptotes—that is, K2–K1, hereafter “asymptotic width”—were used to assess differences between groups.

The identification parameters (boundary, slope, asymptote width) were analyzed with Continuum (ba/pa, de/te, di/ti) × Group (DYS, NR) × Grade (2, 4) repeated‐measures ANOVAs. The Greenhouse–Geisser adjustments were performed when appropriate.

#### 2.3.2 Discrimination data

For each VOT pair (e.g., S1S3) and each participant, correct discrimination scores were calculated by taking the mean of the “different” responses to the different stimulus pairs (e.g., S1S3 and S3S1) and the “same” responses to the pairs including different stimuli (e.g., S1S1 and S3S3). The discrimination scores were converted into *d*‐prime (*d*′) scores by taking the difference between the standard normal deviates (*Z* values) of the same and different pairs (McMillan et al., 1977; for details, see Medina et al., 2010).

Differences between groups were analyzed with VOT (central values of the stimulus pairs used for calculating the discrimination scores: −40, −30, −20, −10, 0, +10, +20, +30, and +40) × Continuum (ba/pa, de/te, di/ti) × Group (DYS, NR) × Grade (2, 4) repeated‐measures ANOVAs. Two planned contrasts were used for analyzing the Pair × Group and Pair × Grade interactions, each corresponding to a possible difference on theoretical grounds, namely (a) the “phonemic peak” contrast, that is, the difference between the discrimination score of the between‐category pair (i.e., the one straddling 0‐ms VOT that was closest to the phonemic boundary) and the mean scores of other pairs (those centered on ±40‐ and −±20‐ms VOT; excluding the ±10‐ms pairs, adjacent to the phonemic boundary that is theoretically located at 0 ms but empirically fluctuates somewhat around this value; also excluding the ±30‐ms pairs straddling the allophonic boundaries); (b) the “allophonic peaks” contrast, that is, the difference between the pairs straddling the ±30‐ms VOT boundaries and the adjacent ones (those centered on −40‐, −20‐, 20‐, and 40‐ms VOT).

## 3 RESULTS

Of the 204 participants in the study, seven displayed flat response curves for at least one of the two tasks (identification and/or discrimination). Among these seven participants, four were affected by dyslexia (two in each Grade) and three were typical readers (one in Grade 2, two in Grade 4). The results of these seven children were not included in the following analyses, which were thus based on 197 cases.

### 3.1 Identification data

**FIGURE 2:**
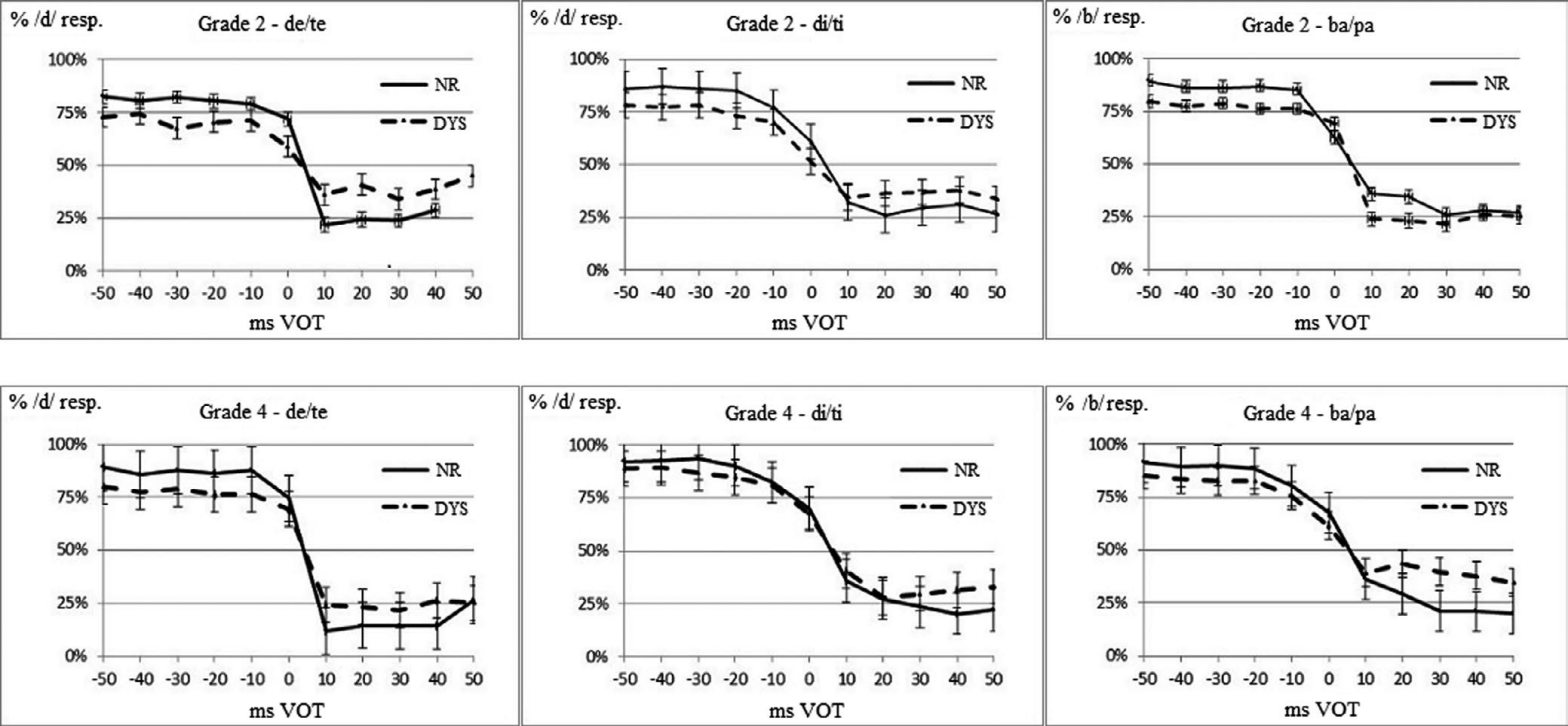
Identification functions of the dyslexic (DYS) and normal‐reading (NR) groups per Grade (top: Grade 2; bottom: Grade 4), and Continuum (left: de/te; middle: di/ti; right: ba/pa)

The identification scores are presented in Figure 2 as a function of Reading Group, Grade, and Continuum. The values of the boundary, slope, and asymptote of the identification functions are presented in Tables 2, 3, 4, respectively.

**TABLE 2:** Phonemic boundary location for each group and each continuum

Phonemic boundary location			
	ba/pa	de/te	di/ti
NR Grade 2	5.7 (17)	−0.2 (13)	4.1 (16)
NR Grade 4	1.4 (14)	3.9 (8.0)	5.3 (12)
DYS Grade 2	7.5 (25)	−0.4 (22)	2.1 (25)
DYS Grade 4	7.6 (21)	2.0 (12)	2.2 (13)
All Groups	5.7 (20)	1.0 (15)	3.4 (18)
General Mean	3.4 (18) different from 0, *p* < .05		

Note

Mean (*SD*) in ms.

Abbreviations: DYS, dyslexic; NR, normal reader.

**TABLE 3:** Asymptote width for each group and each continuum

Asymptote width			
	ba/pa	de/te	di/ti
NR Grade 2	0.74 (0.20)	0.70 (0.23)	0.70 (0.22)
NR Grade 4	0.77 (0.22)	0.78 (0.19)	0.79 (0.17)
DYS Grade 2	0.66 (0.23)	0.68 (0.25)	0.63 (0.24)
DYS Grade 4	0.66 (0.21)	0.76 (0.19)	0.71 (0.21)

Note

Mean (*SD*) in %.

Abbreviations: DYS, dyslexic; NR, normal reader.

**TABLE 4:** Slope for each group and each continuum

Slope			
	ba/pa	de/te	di/ti
NR Grade 2	0.40 (0.44)	0.64 (0.68)	0.56 (0.41)
NR Grade 4	0.37 (0.34)	0.80 (0.35)	0.45 (0.41)
DYS Grade 2	0.32 (0.43)	0.34 (0.66)	0.35 (0.55)
DYS Grade 4	0.49 (0.44)	0.61 (0.56)	0.48 (0.41)

Note

Mean (*SD*) in logit/ms.

Abbreviations: DYS, dyslexic; NR, normal reader.

The mean VOT boundary was located at 3.4 ms (*SD* = 18), and it was significantly different from 0 ms (*t*(590) = 4.59, *p* < .001, *η*² = 0.034). A Group (DYS, NR) × Grade (2, 4) × Continuum (ba/pa, de/te, di/ti) ANOVA did not reveal significant effects (group and Grade: both *F* < 1; Continuum: *F*(2.0, 379) = 2.76, *p* = .07, *η*² = 0.014; interactions: *p* > .15). Figure 2 and Table 2 show that the perceptual boundary was located around 0‐ms VOT irrespective of Reading Group and Grade.

Concerning the asymptotes of the identification function, a Group × Grade × Continuum ANOVA showed that the effects of Group and Grade were significant (*F*(1,193) = 9.10, *p* < .01, *η*² = 0.045; 8.85, *p* < .01, *η*² = 0.044, respectively). Figure 2 and Table 3 show that the asymptote width was larger for the NR than for the DYS, and for Grade 4 compared with Grade 2. The continuum had no significant effect (*F* < 1), and all the interactions were not significant (*p* > .18).

Concerning the slope of the identification function, a Group × Grade × Continuum ANOVA indicated that the main effects of Group, Grade, and Continuum were significant (*F*(1,193) = 5.66, *p* < .05, *η*² = 0.028; 5.31, *p* < .05, *η*² = 0.027; *F*(1.8, 348) = 8.70, *p* < .001, *η*² = 0.043, respectively). The Group × Grade and Group × Continuum interactions were also significant (*F*(1,193) = 4.47, *p* < .05, *η*² = 0.023; *F*(1.8, 348) = 3.43, *p* < .05, *η*² = 0.017, respectively). The other interactions were not significant (*p* > .10). The difference between Reading Groups was significantly larger for the d/t continua than for the ba/pa one (*F*(1,193) = 6.27, *p* < .05, *η*² = 0.031). Figure 2 and Table 4 show that the slope tended to be steeper for the NR than for the DYS, and for the children in Grade 4 compared with those in Grade 2. However, the difference between Reading Groups was smaller for the children in Grade 4 than for those in Grade 2, and it was larger for the de/te and di/ti continua than for the ba/pa one.

In order to control the effect of reading experience, the differences in identification parameters between the DYS group at Grade 4 and the NR group at Grade 2 were tested with Group × Continuum repeated‐measures ANOVAs. For the boundary and for the slope, the main effect of Group and the Group × Continuum interaction was not significant (both *F* < 1). For the asymptote width, the Group effect was not significant (*F* < 1) and the Group × Continuum interaction was marginally significant (*F*(2,198) = 2.88, *p* = .06, *η*² = 0.026). When tested separately with univariate ANOVA for each continuum, the Group effect approached significance for the ba/pa continuum (*F*(1, 99) = 3.28, *p* = .07, *η*² = 0.032).

To sum up, the phonemic boundary was located slightly, but significantly, above the expected 0‐ms VOT value, irrespective of Continuum, Group, and Grade. The asymptote width was smaller for the DYS than for the NR group and also smaller at Grade 2 than at Grade 4. The slope was shallower for the DYS than for the NR group, and the difference between groups was smaller at Grade 4 than at Grade 2 and also smaller for the ba/pa continuum compared with the d/t ones. When reading experience was controlled for, the only group difference that approached significance was the asymptote width along the ba/pa continuum.

### 3.2 Discrimination data

A Group × Grade × VOT × Continuum repeated‐measures ANOVA showed that the VOT × Group × Continuum and VOT × Grade × Continuum interactions were significant (*F*(11.3, 2,090) = 2.7, *p* < .01, *η*² = 0.011; 3.95, *p* < .001, *η*² = 0.021, respectively) and the VOT × Group × Grade × Continuum interaction was not significant (*F* < 1). Separate Group × VOT and Grade × VOT repeated‐measures ANOVAs were then run on the discrimination scores for each continuum.

For the de/te continuum, the Group × VOT interaction was significant (*F*(3.5, 650) = 9.45, *p* < .001, *η*² = 0.047) and the phonemic peak was significantly larger for the NR than for the DYS (Group × VOT planned contrast: *F*(1,191) = 15.4, *p* < .001, *η*² = 0.075); the Grade × VOT interaction was significant (*F*(3.4, 650) = 9.24, *p* < .001, *η*² = 0.046); and the phonemic peak was significantly larger for Grade 4 than for Grade 2 (Group × VOT planned contrast: *F*(1,191) = 16.6, *p *< .001, *η*² = 0.080). For the di/ti continuum, the Group × VOT interaction was significant (*F*(5.9, 1,136) = 3.47, *p* < .001, *η*² = 0.018) and the phonemic peak was significantly larger for the NR than for the DYS (Group × VOT planned contrast: *F*(1,191) = 12.7, *p* < .001, *η*² = 0.062); the Grade × VOT interaction was not significant (*F*(5.9, 1,124) = 1.54, *p* = .16, *η*² = 0.008). For the ba/pa continuum, the Group × VOT interaction was significant (*F*(6.9, 1,343) = 2.67, *p* = .01, *η*² = 0.014), the allophonic peaks were significantly larger for the DYS than for the NR (Group × VOT planned contrast: *F*(1,195) = 6.31, *p* < .05, *η*² = 0.031); the Grade × VOT interaction was not significant (*F*(6.9, 1,343) = 1.22, *p* = .29, *η*² = 0.006).

**FIGURE 3:**
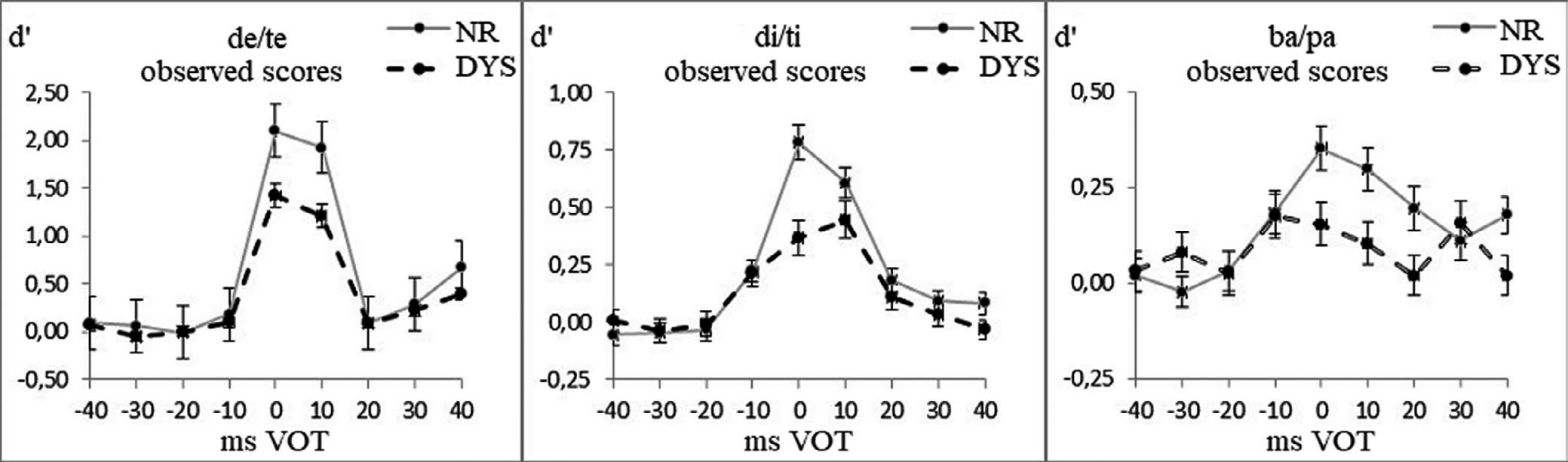
Discrimination functions (*d*′ scores; standard deviation (*SD*)) for the three continua as a function of the Reading Group (data collapsed across both grades per group)

Figure 3 presents the discrimination scores of the DYS and NR groups for each continuum, irrespective of Grade. For the de/te and di/ti continua (Figure 3a,b), the most salient differences between groups resided in the size of the phonemic peak (around 0 VOT), which was larger for the NR than for the DYS. For the ba/pa continuum (Figure 3c), the most salient group differences resided in the fact that discrimination peaks at ±30‐ms VOT were only present for the DYS, not for the CTL, remembering that these peaks refer to the differences between the pairs straddling the ±30‐ms VOT boundaries and the adjacent ones.

In order to control the effect of reading experience, the differences in discrimination scores between the DYS group at Grade 4 and the NR group at Grade 2 were tested with a Group × VOT ANOVAs for each continuum. The Group × Pair interaction was not significant for the de/te and di/ti continua (*F* < 1; *F*(5.9, 577) = 1.05, *p* = .39, *η*² = 0.011, respectively) and marginally significant for the ba/pa continuum (*F*(6.5, 649) = 1.73, *p* = .10, *η*² = 0.017).

To sum up, significant group differences in discrimination for phonemic discrimination peak were observed for the de/te and di/ti continua at 0‐ms VOT, and for the allophonic discrimination peaks for the ba/pa continuum at ±30‐ms VOT. When reading experience was controlled for, differences in discrimination between groups were not significant, although there was a trend for the ba/pa continuum.

### 3.3 Fitting a neural‐based model to discrimination scores

**FIGURE 4:**
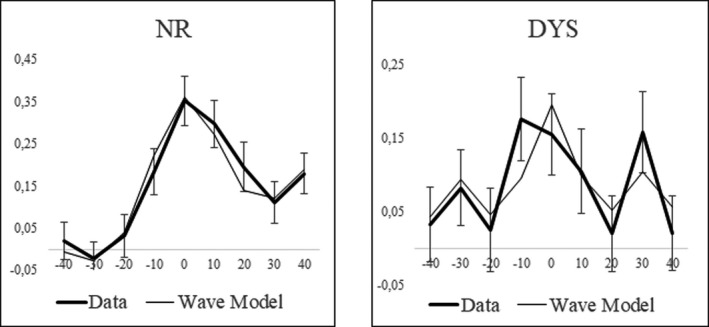
Fitting of the discrimination scores (*d*′; *SD*) for the ba/pa continuum with the network model

A neural‐based model (Equation 2) was fitted to the ba/pa discrimination curve of the NR and DYS groups (data collapsed across both grades per group). With this equation, discrimination scores are conceived as a weighted sum of a 33‐Hz oscillator that captures the sensitivity to allophonic VOT contrasts (at ±30 ms) and of a 17‐Hz oscillator (binary subharmonic of 33 Hz) that captures the sensitivity to a phonemic contrast centered on 0‐ms VOT. We added a linear component to take account of the increased discrimination of stimulus differences in the positive VOT region (Figure 4), an effect that is not specific to the present data (Hoonhorst, Colin, et al., 2009; Medina et al., 2010) and might be due to the covariation of positive VOT with secondary voicing cues.

Equation 2. Neural model. D is a general constant, and C is the weight of the 33‐Hz oscillators.

$$d^{\prime }\left(\text{resp}\right)=\text{VOT}*k+D+C*\cos\left(2\pi *0.033*\text{VOT}\right)+\left(D‐C\right)*\cos\left(2\pi *0.017*\text{VOT}\right).$$

Equation 2 was fitted separately to the ba/pa discrimination responses of each participant, with nonlinear regressions.

Figure 4 demonstrates that the network model fitted the data fairly well. The difference between the discrimination scores predicted by the model and those observed was not significantly different for both groups (Score type × VOT interaction: both *F* < 1). Although the model was quite simple with only three parameters, it follows the overall profile of the discrimination curves for both groups.

However, there were a highly significant between‐group difference in the weight of the 17‐Hz oscillator (D–C: *z* = 3.24, *p* = 001) and no significant difference in the weight of the 33‐Hz oscillator (C: *z* = 1.04, *p* = .30). The weight of the 17‐Hz oscillator was significantly different from 0 for both groups (*Z* = 10.2; 5.27, for the NR and DYS, respectively, both *p* < .001). However, the weight of the 33‐Hz oscillator was significantly different from 0 for the DYS group, but not for the NR group (*Z* = 2.73, *p* < .01; *Z* = 1.36, *p* = .09, respectively). The weight of the 33‐Hz oscillator was significantly lower than the one of the 17‐Hz one for the NR group (*Z* = 6.39, *p* < .001), whereas the difference between weights was only marginally significant for the DYS (*z* = 1.87, *p* = .06). Finally, the slope of the linear component was significantly larger for the NR than for the DYS (*Z* = 2.38, *p* < .05) and it was significantly different from 0 for the NR, but not for the DYS (*Z* = 3.71, *p* < .001; *Z* = 0.24, *p* = .41, respectively).

To sum up, the 17‐Hz oscillator, with only a faint contribution from the 33‐Hz oscillators, fitted the discrimination curve of the NR group fairly well. By contrast, the 17‐ and 33‐Hz oscillators contributed with fairly equivalent weights to fit the discrimination curve of the DYS group. Adding a linear component contributed to improve the model for the NR but not for the DYS.

Fitting the data with a 17‐ to 33‐Hz neural model suggests that, compared with the control group, the children with dyslexia exhibited a weaker degree of subharmonic coupling between 33‐Hz oscillators characterized by a weaker sensitivity to lower frequency oscillations (17 Hz) and a stronger sensitivity to higher frequency oscillations (33 Hz).

**FIGURE 5:**
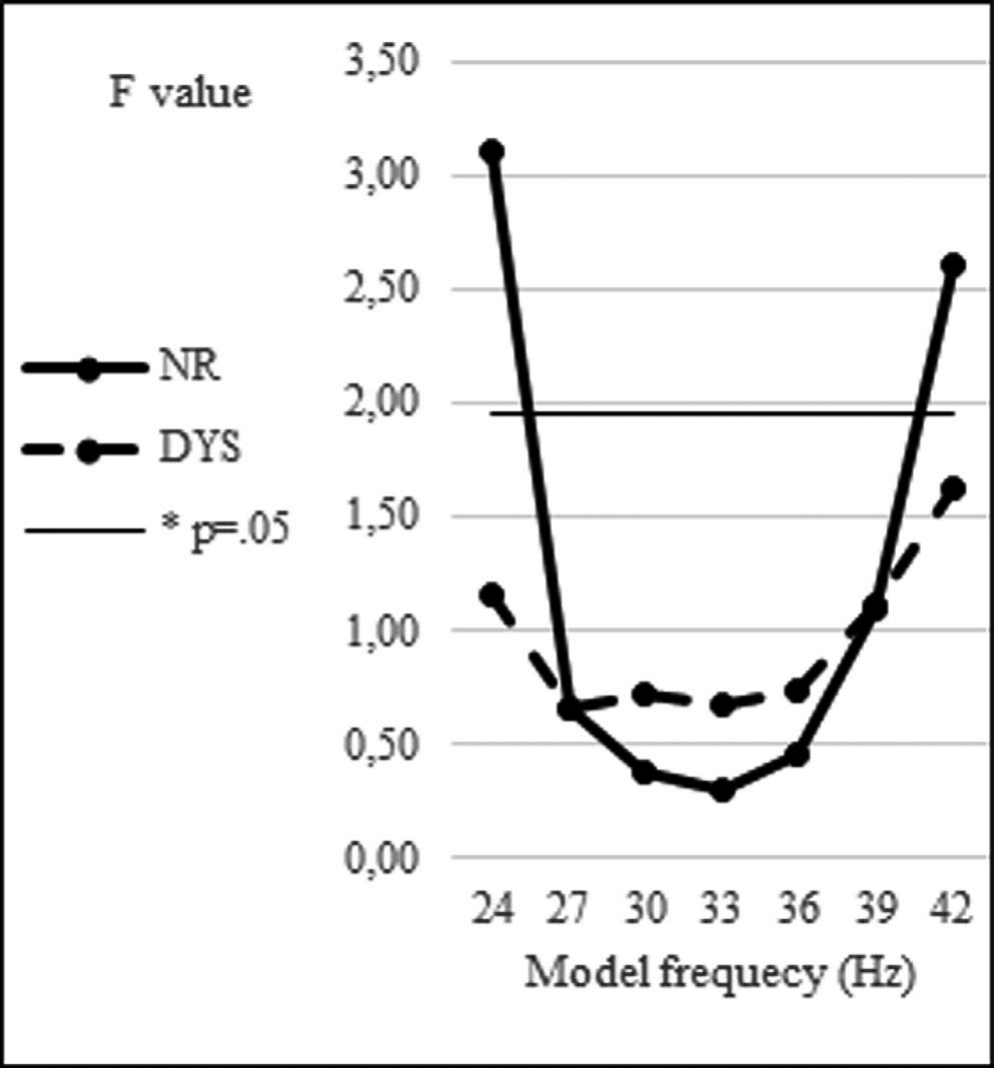
Performances of different models operating in a range of different frequencies and indexed in *F* values of the Score type (Model, Data) × VOT interaction, separately for the NR and DYS groups (*: line corresponding to *p* = .05). Frequencies reported on the abscissa were associated with their subharmonics

Finally, the performance of the 33‐ to 17‐Hz model is fairly optimal compared with alternative models based on oscillators operating at slightly different frequencies. The frequencies of the control values were those corresponding to a set of values sufficiently large to evidence a peak around 33 Hz (and its 17‐Hz subharmonic). Figure 5 gives the performances of different models operating in a range of different frequencies around 33 Hz, with corresponding subharmonics operating around 17 Hz. Frequencies reported on this Figure were associated with their subharmonics (e.g., 36 Hz with 18 Hz, etc.).

The performances are indexed in *F* values of the Score type (Model, Data) × VOT ANOVA interaction, a lower F score indicating a better fit. For the NR group, the performance is fairly stable in a frequency region around 33 Hz and decreases sharply outside this region. For the DYS group, the performance is also fairly stable for the oscillator frequencies in the same region around 33 Hz but decreases more progressively outside this region. Such comparisons suggest that the choice of a 33 Hz as oscillator frequency for modeling VOT perception has some validity and they point to the high‐beta/low‐gamma range as a guideline for further investigations.

## 4 DISCUSSION

Our objective in the empirical study was to assess deficits in the perception of the voicing feature in Spanish children with dyslexia. Different perceptual deficits were assessed with stimuli varying on three different VOT continua in a fairly large sample of children. The results indicated that the stimulus continuum played an important role in defining these differences. The shallower slope around the phonemic boundary (in the middle of the continuum) demonstrated the deficit in categorical *precision*, which is characterized by a smaller difference in category labeling at the continuum endpoints. Also, a smaller phonemic discrimination peak (around the boundary) was more evident for the de/te and di/ti continua than for the ba/pa continuum. Sensitivity to allophonic boundaries was only evidenced for the ba/pa continuum, and not for the two other continua.

### 4.1 Differences in perceptual deficits between continua

To explain the effect of the continuum on the relative salience of the perceptual deficits, one should first remember that the present results evidenced differences in categorical precision between continua irrespective of group (reading status and grade). The slope of the identification curve and the size of the phonemic peak were larger for the d/t continua than for the ba/pa one, and for the de/te continuum than for the di/ti one. Such differences might be related to lexical factors. Both /ba/ and /pa/ are pseudowords in Spanish, whereas the /de/, /te/, /di/, and /ti/ are real words. The perception of speech sounds depends on their lexical status. Minimal contrasts between words, differing as a function of single distinctive feature, are perceived with a better precision than minimal contrasts between pseudowords (Bouton et al., 2012). Accordingly, the fact that categorical precision was lower for the ba/pa continuum than for the two d/t continua can be attributed to a difference in lexical status. Remember that both /ba/ and /pa/ are pseudowords in Spanish, whereas the /de/, /te/, /di/, and /ti/ are real words. In addition to lexical status, the frequency of occurrence may affect the categorical precision. Categorical precision was larger for the de/te continuum than for the di/ti one, which may be because in Spanish the frequency of occurrence for both /di/ and /ti/ (284 and 577 occurrences on LEXESP; Sebastián et al., 2000) is well below both /de/ and /te/ (264,721 and 5,026 occurrences on LEXESP).

The specific nature of the lexical effects on categorical precision remains unknown. One possible explanation is that word contrasts are perceived with a higher degree precision because that have been heard more frequently before, irrespective of the reading status of the listener.

### 4.2 Effects of reading status on the precision of the phonemic boundary

The presence of a categorical precision deficit in Spanish children with dyslexia extends the results of previous studies that evidenced such deficit across various other languages, including Chinese, Dutch, English, and French. A meta‐analysis based on the results of 36 studies evidenced a reliable categorical precision deficit in individuals with dyslexia (Noordenbos & Serniclaes, 2015). A mean Cohen's *d* effect size of 0.86 (C.I.: [0.56–1.16] was found for the differences between DYS and NR age‐matched controls based on the magnitude of the phonemic discrimination peak. In the present study, the effect size based on the discrimination scores and calculated from the Phonemic Peak × Group interaction (for the NR age controls and the three continua taken together) amounts to 0.67. The categorical precision deficit that was found here for Spanish children with dyslexia therefore falls in the range of those that have been documented in other languages.

### 4.3 Sensitivity to allophonic boundaries

Allophonic perception was only evidenced for the ba/pa continuum, in which the categorical precision was smallest. Children with dyslexia exhibited an enhanced sensitivity to the allophonic VOT boundaries (Figure 2c). Following allophonic theory, people with dyslexia should be sensitive to universal VOT boundaries, at −30 ms and +30 ms, different from the phonological VOT boundary that is located at 0 ms in languages such as Spanish and French. In the present study, the Spanish children with dyslexia exhibited this sensitivity for the ba/pa continuum (Figure 3). This is the first time that an enhanced sensitivity is evidenced for both allophonic VOT boundaries. Previous studies, conducted with French children with dyslexia and with other VOT continua, only evidenced an allophonic sensitivity to the −30‐ms VOT boundary (Bogliotti et al., 2008; Serniclaes et al., 2004). The fact that the phonemic boundary was located inside the positive VOT region on these continua may have masked the enhanced sensitivity of the dyslexic children to the +30‐ms VOT boundary in these studies.

The lack of significant difference in behavioral discrimination responses between DYS and younger NR of the same reading age is in accordance with previous results showing that allophonic perception with behavioral data depends on the age/grade of the children. In a follow‐up study with Dutch children with a familial risk for dyslexia, sensitivity to an allophonic place‐of‐articulation boundary was demonstrated with behavioral data when these children were at Grade 1. This sensitivity was no longer present in the behavioral responses of the children when they were at Grade 2, but it was still present in neurophysiological recordings (Noordenbos et al., 2012b). These results highlight the evanescent character of allophonic perception in behavioral responses despite their persistence at the neural level. Reading experience seemingly has an inhibitory effect on the behavioral manifestations of allophonic perception, and this might explain the lack of difference between DYS and NR controls in the present study. Also, the fact that allophonic perception was only evidenced for the ba/pa continuum, not for the de/te and di/ti continua, is probably due to the difference in lexical status between these continua. Lexicality seemingly also had inhibitory effect on the behavioral manifestations of allophonic perception.

### 4.4 Neural modeling

Fitting the data with a subharmonic coupling model suggests that children with dyslexia are more sensitive than controls to ~33‐Hz oscillations, appropriate to the perception of universal VOT boundaries at ±30‐ms VOT, and less sensitive than controls to lower frequency oscillations (at ~17 Hz), appropriate to the perception of Spanish boundary at 0‐ms VOT (Figure 4). This is compatible with a neural interpretation of allophonic perception that attributes the increased sensitivity to universal features to a weaker coupling between high‐frequency oscillators.

This neural model is also compatible with the “temporal sampling” (TS) theory that conceives speech perception as a hierarchical process, with lower frequency oscillators controlling the phase relationships between higher ones (Goswami, 2011, 2019). Both TS theory and the present model converge to attribute phonological dyslexia to a coupling deficit between oscillators, although there are some differences in the way they conceive phoneme perception. According to TS, phoneme perception is a matter of phase locking relatively low‐frequency neural oscillations (in the delta–theta range) with acoustic oscillations that delimitate phonemic segments in the speech signal, and allophonic perception arises from the hypersegmentation of the acoustic signal (Lehongre et al., 2011). The present model considers that phoneme perception is achieved by subharmonic coupling between different neural oscillators operating at relatively high frequencies (beta and low‐gamma range). According to new empirical and simulation advances, beta–gamma couplings would play a crucial role on speech recognition (e.g., Hovsepyan et al., 2020; Pefkou et al., 2017), and hypersegmentation results from a lack of inhibition of high‐frequency oscillators. In this view, the enhanced activity above 50 Hz that was evidenced in a study of French adults with dyslexia (Lehongre et al., 2011) would reflect the activity of a relatively dense network of short‐phased oscillators (as illustrated in Figure 1b).

## 5 CONCLUSIONS

Previous results on allophonic perception of voicing contrasts in French were confirmed and cross‐validated with data on Spanish. Allophonic perception was only evidenced for one of the three VOT continua under scope (ba/pa), presumably because it was based on a nonlexical contrast. However, the role of lexical factors for evidencing allophonic sensitivity to allophonic distinctions needs to be confirmed with systematic comparisons between several lexical and nonlexical contrasts. The present data also give preliminary support to the role subharmonic coupling between oscillators in VOT perception, a contention that should be further investigated with brain data.

## CONFLICT OF INTEREST

None declared.

## AUTHOR CONTRIBUTIONS

Willy Serniclaes: Conceptualization; supervision; methodology; writing—original draft preparation; and formal analysis. Miguel López‐Zamora: Software; investigation; visualization; and writing—review and editing. Soraya Bordoy: Investigation. Juan L. Luque: Corresponding author; conceptualization; resources; investigation; funding acquisition; and writing—review and editing.

## ETHICAL APPROVAL

This research does not require an ethical statement or evaluation.

### PEER REVIEW

The peer review history for this article is available at https://publons.com/publon/10.1002/brb3.2194.

## Data Availability

Data openly available in a public repository that issues datasets with DOIs. Repository: https://osf.io/e2n7z/?view_only=e7bb09e8536d4b109bebd98aa5d4b7c4.

## Footnotes

^1^ In French, /də/ frequency amounts to 5,607,822 against 812,928 for /pa/, the most frequent in other studies; Brulex database; Content et al., 1990.

## References

[brb32194-bib-0001] Abramson, A. S. (1977). Laryngeal timing and consonant distinctions. Phonetica, 34, 295–303. 594164 10.1159/000259888

[brb32194-bib-0002] Abramson, A. S. , & Lisker, L. (1970). Discriminability along the voice onset time continuum: cross‐language tests. In B. Hala , M. Romportl , & P. Janota (Eds.), Proceedings of the 6th international congress of phonetic sciences, Prague 1967 (pp. 569–573). Academia.

[brb32194-bib-0003] Abramson, A. S. , & Lisker, L. (1973). Voice‐timing perception in Spanish word‐initial stops. Journal of Phonetics, 1, 1–8. 10.1016/S0095-4470(19)31372-5

[brb32194-bib-0004] Bogliotti, C. , Serniclaes, W. , Messaoud‐Galusi, S. , & Sprenger‐Charolles, L. (2008). Discrimination of speech sounds by dyslexic children: Comparisons with chronological age and reading level controls. Journal of Experimental Child Psychology, 101, 137–175. 18462745 10.1016/j.jecp.2008.03.006

[brb32194-bib-0005] Bordoy, S. (2015). De la teoría fonológica a la identificación temprana y el diagnostico diferencial de la dislexia evolutiva. [From phonological theory to early identification and differential diagnostic of developmental dyslexia] PhD Thesis. Departamento de Psicología Evolutiva y de la Educación Doctorado en Psicología; Universidad de Málaga. Spain.

[brb32194-bib-0006] Bouton, S. , Serniclaes, W. , Colé, P. , & Bertoncini, J. (2012). Perception of speech features by French‐speaking children with cochlear implant. Journal of Speech, Language, and Hearing Research, 55, 139–153. 10.1044/1092-4388(2011/10-0330) 22199195

[brb32194-bib-0008] Chomsky, N. , & Halle, M. (1968). The sound pattern of English. Harper and Row.

[brb32194-bib-0009] Content, A. , Mousty, P. , & Radeau, M. (1990). Brulex: Une base de données lexicales informatisée pour le Français écrit et parlé. Année Psychologique, 90, 551–566. 10.3406/psy.1990.29428

[brb32194-bib-0010] Goswami, U. (2011). A temporal sampling framework for developmental dyslexia. Trends in Cognitive Sciences, 15, 1–10. 10.1016/j.tics.2010.10.001 21093350

[brb32194-bib-0011] Goswami, U. (2019). A neural oscillations perspective on phonological development and phonological processing in developmental dyslexia. Language Linguistic Compass, 13, e12328. 10.1111/lnc3.12328

[brb32194-bib-0012] Hämäläinen, J. , Landi, N. , Loberg, O. , Lohvansuu, K. , Pugh, K. , & Leppänen, P. H. T. (2018). Brain event‐related potentials to phoneme contrasts and their correlation to reading skills in school‐age children. International Journal of Behavioral Development, 42, 357–372. 10.1177/0165025417728582 29892138 PMC5992924

[brb32194-bib-0014] Hickok, G. , & Poeppel, D. (2007). The cortical organization of speech processing. Nature reviews Neuroscience, 8, 393–402. 10.1038/nrn2113 17431404

[brb32194-bib-0015] Hoonhorst, I. , Colin, C. , Markessis, E. , Radeau, M. , Deltenre, P. , & Serniclaes, W. (2009). French native speakers in the making: From language‐general to language‐specific voicing boundaries. Journal of Experimental Child Psychology, 104, 353–366. 10.1016/j.jecp.2009.07.005 19709671

[brb32194-bib-0016] Hoonhorst, I. , Medina, V. , Colin, C. , Markessis, E. , Radeau, M. , Deltenre, P. , & Serniclaes, W. (2011). The development of categorical perception: Comparisons between voicing, colors and facial expressions. Speech Communication, 53, 417–430. 10.1016/j.specom.2010.11.005

[brb32194-bib-0017] Hoonhorst, I. , Serniclaes, W. , Collet, G. , Colin, C. , Markessis, E. , Radeau, M. , & Deltenre, P. (2009). The acoustic correlates of voicing perception in French. Clinical Neurophysiology, 120, 897–903. 19329357 10.1016/j.clinph.2009.02.174

[brb32194-bib-0018] Hovsepyan, S. , Olasagasti, I. , & Giraud, A. L. (2020). Combining predictive coding and neural oscillations enables online syllable recognition in natural speech. Nature Communications, 11(1), 1–12. 10.1038/s41467-020-16956-5 PMC7305192 32561726

[brb32194-bib-0019] Jakobson, R. , Fant, G. , & Halle, M. (1952). Preliminaries to speech analysis. The distinctive features and their correlates. M.I.T. Press.

[brb32194-bib-0020] Langdon, A. J. , Boonstra, T. W. , & Breakspear, W. (2011). Multi‐frequency phase locking in human somatosensory cortex. Progress in Biophysics and Molecular Biology, 105, 58–66. 10.1016/j.pbiomolbio.2010.09.015 20869386

[brb32194-bib-0021] Lehongre, K. , Ramus, F. , Villiermet, N. , Schwartz, D. , & Giraud, A.‐L. (2011). Altered low‐gamma sampling in auditory cortex accounts for the three main facets of dyslexia. Neuron, 72, 1080–1090. 10.1016/j.neuron.2011.11.002 22196341

[brb32194-bib-0022] Li, M. Y. C. , Braze, D. , Kukona, A. , Johns, C. L. , Tabor, W. , Van Dyke, J. A. , Mencl, W. E. , Shankweiler, D. P. , Pugh, K. R. , & Magnuson, J. S. (2019). Individual differences in subphonemic sensitivity and phonological skills. Journal of Memory and Language, 10.1016/j.jml.2019.03.008 PMC6701851 31431796

[brb32194-bib-0023] Lisker, L. , & Abramson, A. S. (1964). A cross‐language study of voicing in initial stops: Acoustical measurements. Word, 20, 384–422. 10.1080/00437956.1964.11659830

[brb32194-bib-0024] Lisker, L. , & Abramson, A. S. (1967). Some effects of context on voice onset time in English stops. Language and Speech, 10, 1–28. 10.1177/002383096701000101 6044530

[brb32194-bib-0025] Lisker, L. , & Abramson, A. S. (1970). The voicing dimension: some experiments in comparative phonetics. In Proceedings of the 6th international congress of phonetic sciences, Prague 1967 (pp. 563–567); Academia.

[brb32194-bib-0026] Lisker, L. , Liberman, A. M. , Erickson, D. M. , & Dechovitz, D. (1977). On pushing the voice onset time boundary about. Language and Speech, 20, 209–216. 613182 10.1177/002383097702000303

[brb32194-bib-0027] Macmillan, N. A. , Kaplan, H. L. , & Creelman, C. D. (1977). The psychophysics of categorical perception. Psychological Review, 84(5), 452–471. 10.1037/0033-295X.84.5.452 905471

[brb32194-bib-0028] Marín, J. , & Carrillo, M. S. (1997). Test de Eficiencia Lectora‐TECLE. In A. Cuadro , D. Costa , D. Trias & P. Ponce de León (Eds.), Evaluación del nivel lector. Manual técnico del test de Eficacia Lectora (TECLE) (pp. 247–248). Prensa Médica Latinoamericana.

[brb32194-bib-0030] Medina, V. , Hoonhorst, I. , Bogliotti, C. , & Serniclaes, W. (2010). Development of voicing perception in French: Comparing adults, adolescents and children. Journal of Phonetics, 36, 493–503. 10.1016/j.wocn.2010.06.002

[brb32194-bib-0031] Melby‐Lervåg, M. , Lyster, S. A. H. , & Hulme, C. (2012). Phonological skills and their role in learning to read: A meta‐analytic review. Psychological Bulletin, 138, 322–352. 10.1037/a0026744 22250824

[brb32194-bib-0032] Noordenbos, M. W. , Segers, E. , Serniclaes, W. , Mitterer, H. , & Verhoeven, L. (2012a). Allophonic mode of speech perception in Dutch children at risk for dyslexia: A longitudinal study. Research in Developmental Disabilities, 33, 1469–1483. 10.1016/j.ridd.2012.03.021 22522205

[brb32194-bib-0033] Noordenbos, M. W. , Segers, E. , Serniclaes, W. , Mitterer, H. , & Verhoeven, L. (2012b). Neural evidence of allophonic perception in children at risk for dyslexia. Neuropsychologia, 50, 2010–2017. 10.1016/j.neuropsychologia.2012.04.026 22569214

[brb32194-bib-0034] Noordenbos, M. W. , Segers, E. , Serniclaes, W. , & Verhoeven, L. (2013). Neural evidence of the allophonic mode of speech perception in adults with dyslexia. Clinical Neurophysiology, 124, 1151–1162. 10.1016/j.clinph.2012.12.044 23403261

[brb32194-bib-0035] Noordenbos, M. , & Serniclaes, W. (2015). Categorical perception in dyslexia: A meta‐analysis. Scientific Studies of Reading, 19, 340–359. 10.1080/10888438.2015.1052455

[brb32194-bib-0036] O’Brien, G. E. , McCloy, D. R. , Kubota, E. C. , & Yeatman, J. D. (2018). Reading ability and phoneme categorization. Scientific Reports, 8, Article 16842. 10.1038/s41598-018-34823-8 30442952 PMC6237901

[brb32194-bib-0037] Pefkou, M. , Arnal, L. H. , Fontolan, L. , & Giraud, A. L. (2017). θ‐Band and β‐band neural activity reflects independent syllable tracking and comprehension of time‐compressed speech. Journal of Neuroscience, 37(33), 7930–7938. 10.1523/JNEUROSCI.2882-16.2017 28729443 PMC6596908

[brb32194-bib-0038] Peterson, R. L. , & Pennington, B. F. (2012). Developmental dyslexia. Lancet, 379, 1997–2007. 10.1016/S0140-6736(12)60198-6 22513218 PMC3465717

[brb32194-bib-0039] Pisoni, D. B. (1977). Identification and discrimination of the relative onset time of two components tones: Implications for voicing perception in stops. Journal of the Acoustical Society of America, 61, 1352–1361. 10.1121/1.381409 881488

[brb32194-bib-0040] Raven, J. , Raven, J. C. , & Court, J. H. (1996). Raven, matrices progresivas. Tea Ediciones.

[brb32194-bib-0041] Richards, F. J. (1959). A flexible growth model for empirical use. Journal of Experimental Botany, 10, 290–300.

[brb32194-bib-0042] Roberts, J. A. , & Robinson, P. A. (2012). Quantitative theory of driven nonlinear brain dynamics. NeuroImage, 62, 1947–1955. 10.1016/j.neuroimage.2012.05.054 22652022

[brb32194-bib-0043] Rufener, K. S. , Krauel, K. , Meyer, M. , Heinze, H. J. , & Zaehle, T. (2019). Transcranial electrical stimulation improves phoneme processing in developmental dyslexia. Brain Stimulation, 12, 930–937. 10.1016/j.brs.2019.02.007 30826318

[brb32194-bib-0044] Rufener, K. S. , Zaehle, T. , Oechslin, M. S. , & Meyer, M. (2016). 40 Hz‐Transcranial alternating current stimulation (tACS) selectively modulates speech perception. International Journal of Psychophysiology, 101, 18–24. 10.1016/j.ijpsycho.2016.01.002 26779822

[brb32194-bib-0045] Saksida, A. , Iannuzzi, S. , Bogliotti, C. , Chaix, Y. , Démonet, J.‐F. , Bricout, L. , Billard, C. , Nguyen‐Morel, M.‐A. , Le Heuzey, M.‐F. , Soares‐Boucaud, I. , George, F. , Ziegler, J. C. , & Ramus, F. (2016). Phonological skills, visual attention span, and visual stress in developmental dyslexia. Developmental Psychology, 52, 1503–1516. 10.1037/dev0000184 27690491

[brb32194-bib-0046] Sebastián, N. , Martí, M. A. , Carreiras, M. F. , & Cuetos, F. (2000). LEXESP, léxico informatizado del Español. Ediciones de la Universitat de Barcelona.

[brb32194-bib-0047] Serniclaes, W. (1975). Perceptual processing of acoustic correlates of the voicing feature. In: G. Fant (Ed.), Proc. of the Speech Com. Seminar, Stockholm 1974, pp. 87–94.Almqvist & Wiksell.

[brb32194-bib-0048] Serniclaes, W. (1987). Etude expérimentale de la perception du trait de voisement des occlusives du français. [Experimental study of voicing perception in French stop consonants] Thèse de doctorat en Sciences psychologiques. Unpublished doctoral thesis. Université Libre de Bruxelles. Retrieved from http://lpp.psycho.univ‐paris5.fr/person.php?name=WillyS

[brb32194-bib-0049] Serniclaes, W. (2018). Allophonic theory of dyslexia: A short overview. JSM Communication Disorders, 2, 1010.

[brb32194-bib-0050] Serniclaes, W. , & Sprenger‐Charolles, L. (2015). Reading impairment: From behavior to brain. In R. Bahr , & E. Silliman (Eds.), Handbook of Communication Disorders (pp. 34–45). Routledge.

[brb32194-bib-0051] Serniclaes, W. , Sprenger‐Charolles, L. , Carré, R. , & Démonet, J. F. (2001). Perceptual discrimination of speech sounds in developmental dyslexia. Journal of Speech, Language, and Hearing Research, 44, 384–399. 10.1044/1092-4388(2001/032) 11324660

[brb32194-bib-0052] Serniclaes, W. , Van Heghe, S. , Mousty, P. , Carré, R. , & Sprenger‐Charolles, L. (2004). Allophonic mode of speech perception in dyslexia. Journal of Experimental Child Psychology, 87, 336–361. 15050458 10.1016/j.jecp.2004.02.001

[brb32194-bib-0053] Shaywitz, S. E. , & Shaywitz, B. A. (2003). Dyslexia (specific reading disability). Pediatrics in Review, 24(5), 147–153. 12728187 10.1542/pir.24-5-147

[brb32194-bib-0054] Simon, C. , & Fourcin, A. J. (1978). Cross‐language study of speech‐pattern learning. Journal of the Acoustical Society of America, 63, 925–935.

[brb32194-bib-0055] Snowling, M. J. , Lervåg, A. , Nash, H. M. , & Hulme, C. (2019). Longitudinal relationships between speech perception, phonological skills and reading in children at high‐risk of dyslexia. Developmental Science, 22, e12723. 10.1111/desc.12723 30207641 PMC6492008

[brb32194-bib-0056] Steinschneider, M. , Schroeder, C. E. , Arezzo, J. C. , & Vaughan, H. G. Jr (1995). Physiologic correlates of the voice onset time boundary in primary auditory cortex (A1) of the awake monkey: Temporal response patterns. Brain and Language, 48, 326–340. 7757450 10.1006/brln.1995.1015

[brb32194-bib-0057] Steinschneider, M. , Volkov, I. O. , Noh, M. D. , Garell, P. C. , & Howard, M. A. (1999). Temporal encoding of the voice onset time phonetic parameter by field potentials recorded directly from human auditory cortex. Journal of Neurophysiology, 82, 2346–2357. 10561410 10.1152/jn.1999.82.5.2346

[brb32194-bib-0058] Werker, J. F. , & Tees, R. C. (1987). Speech perception in severely disabled and average reading children. Canadian Journal of Experimental Psychology, 41, 48–61. 10.1037/h0084150 3502888

[brb32194-bib-0059] Yang, Y. , Solis Escalante, T. , van der Helm, F. , & Schouten, A. (2016). A generalized coherence framework for detecting and characterizing nonlinear interactions in the nervous system. IEEE Transactions on Biomedical Engineering, 63, 2629–2637. 10.1109/TBME.2016.2585097 27362753

[brb32194-bib-0060] Zaehle, T. , Jancke, L. , & Meyer, M. (2007). Electrical brain imaging evidences left auditory cortex involvement in speech and non‐speech discrimination based on temporal features. Behavioral and Brain Functions, 3(63). 10.1186/1744-9081-3-63 PMC2231369 18070338

[brb32194-bib-0061] Zoubrinetzky, R. , Collet, G. , Nguyen Morel, M.‐A. , Valdois, S. , & Serniclaes, W. (2019). Remediation of allophonic perception and visual attention span in developmental dyslexia: A joint assay. Frontiers in Psychology‐Developmental Psychology. 10.3389/fpsyg.2019.01502 PMC6647912 31379640

